# Derivation of a Clinical Risk Score to Predict 14-Day Occurrence of Hypoxia, ICU Admission, and Death Among Patients with Coronavirus Disease 2019

**DOI:** 10.1007/s11606-020-06353-5

**Published:** 2020-12-03

**Authors:** David M. Levine, Stuart R. Lipsitz, Zoe Co, Wenyu Song, Patricia C. Dykes, Lipika Samal

**Affiliations:** 1 Division of General Internal Medicine and Primary Care, Brigham and Women’s Hospital, Boston, MA USA; 2 Harvard Medical School, Boston, MA USA

**Keywords:** COVID-19, severe acute respiratory syndrome coronavirus 2, patient discharge, prognosis, risk score

## Abstract

**Background:**

Uncertainty surrounding COVID-19 regarding rapid progression to acute respiratory distress syndrome and unusual clinical characteristics make discharge from a monitored setting challenging. A clinical risk score to predict 14-day occurrence of hypoxia, ICU admission, and death is unavailable.

**Objective:**

Derive and validate a risk score to predict suitability for discharge from a monitored setting among an early cohort of patients with COVID-19.

**Design:**

Model derivation and validation in a retrospective cohort. We built a manual forward stepwise logistic regression model to identify variables associated with suitability for discharge and assigned points to each variable. Event-free patients were included after at least 14 days of follow-up.

**Participants:**

All adult patients with a COVID-19 diagnosis between March 1, 2020, and April 12, 2020, in 10 hospitals in Massachusetts, USA.

**Main Measures:**

Fourteen-day composite predicting hypoxia, ICU admission, and death. We calculated a risk score for each patient as a predictor of suitability for discharge evaluated by area under the curve.

**Key Results:**

Of 2059 patients with COVID-19, 1326 met inclusion. The 1014-patient training cohort had a mean age of 58 years, was 56% female, and 65% had at least one comorbidity. A total of 255 (25%) patients were suitable for discharge. Variables associated with suitability for discharge were age, oxygen saturation, and albumin level, yielding a risk score between 0 and 55. At a cut point of 30, the score had a sensitivity of 83% and specificity of 82%. The respective *c*-statistic for the derivation and validation cohorts were 0.8939 (95% CI, 0.8687 to 0.9192) and 0.8685 (95% CI, 0.8095 to 0.9275). The score performed similarly for inpatients and emergency department patients.

**Conclusions:**

A 3-item risk score for patients with COVID-19 consisting of age, oxygen saturation, and an acute phase reactant (albumin) using point of care data predicts suitability for discharge and may optimize scarce resources.

**Supplementary Information:**

The online version contains supplementary material available at 10.1007/s11606-020-06353-5.

## INTRODUCTION

Severe acute respiratory syndrome coronavirus 2 (SARS-CoV-2), the cause of coronavirus disease 2019 (COVID-19), is a pandemic that has infected 20 million individuals and caused over 740,000 deaths worldwide as of August, 2020.^1^ In the USA, COVID-19 is projected to cause over 300,000 deaths by the end of 2020.^2^

COVID-19 presents with a variable array of symptoms^3–6^ and has a highly variable effect on morbidity and mortality,^7^ making the disease a challenge to appropriately triage and manage.^8^ It is estimated that a large percentage of patients are asymptomatic,^9–12^ while about 15% become so ill that they require intensive care.^13^ Reports of rapid decompensation requiring intubation among patients who are otherwise young and healthy further cloud triage and management decisions and may lead clinicians to rely on anecdote or recency bias.^14^

The swift uptick in COVID-19 cases has put a severe strain on healthcare resources in various hotspot regions. Given resource constraints and the unpredictability of the COVID-19 disease trajectory, clinicians face a large challenge in deciding whether a patient can be discharged based on future clinical trajectory.

To aid clinicians at the point of care with disposition decisions surrounding COVID-19 in the emergency department and inpatient ward, we present the derivation and validation of a data-driven clinical risk score to predict the 14-day occurrence of hypoxia, ICU admission, and death.

## METHODS

### Data Source

We extracted structured data from the common electronic health record (EHR) of Mass General Brigham (previously Partners HealthCare) institutions, a consortium of 10 hospitals and clinics in eastern Massachusetts.

The study protocol was deemed exempt by the Mass General Brigham institutional review board and was registered at clinicaltrials.gov (NCT04339387). We followed the Transparent Reporting of a Multivariable Prediction Model for Individual Prognosis or Diagnosis (TRIPOD)^15^ reporting guideline from the Enhancing the Quality and Transparency of Health Research (EQUATOR) Network.^16^

### Participants

We included all patients age 18 years and older who had at least one positive reverse transcription polymerase chain reaction test for SARS-CoV-2. Criteria for testing evolved rapidly at Mass General Brigham offered at first only to seriously ill patients and healthcare workers, and then slowly liberalized (eTable 1). We included patients with a new diagnosis beginning March 1, 2020, until April 12, 2020, when we passed our target enrollment of 1250 patients.

### Outcome

We sought to study whether a patient with COVID-19 was suitable for discharge from a monitored setting. We developed a 14-day composite that predicted the need for supplemental oxygen, need for the intensive care unit (ICU) level of care, and death. We chose this composite endpoint, as opposed to that used in numerous case studies (mechanical ventilation, ICU, and/or death) to be highly clinically relevant in a scenario where inpatient beds are scarce, or barriers existed to sending patients home with oxygen. Our goal was to develop a risk score that is most useful for clinicians in the emergency department or those on the inpatient ward who are making decisions about discharge to home. Because patients present at different phases in their illness, we used a patient’s most recent data to guide management, as is done by clinicians determining a patient’s suitability for discharge.

We opted for a 14-day follow-up period from time of presentation for patients who did not experience this composite endpoint. New research suggests that the median time to symptoms is 8 days and that 90% of patients develop symptoms within 14 days.^17^ In addition, we were not able to ascertain date of symptom onset. “Day zero” was defined as the date of either a positive SARS-CoV-2 test or presentation for evaluation, whichever was earlier, allowing additional lead time.

### Predictors

We a priori chose to focus on predictors that were objective, were readily available, and required little computation so that a risk score could be calculated by hand at the point of care. Though electronic health records and mobile apps allow clinicians to access sophisticated risk prediction models, these can be challenging to integrate, are not available in underserved settings, and do not afford transparency. We used reports from the literature to direct us toward predictors that appeared promising.^4, 7, 18, 19^ The risk score is meant to be used in real time when a patient is assessed for discharge from the emergency department or the inpatient ward, so we chose to use the last recorded measurement for each variable. We extracted age, sex, smoking status (ever-smoker), comorbidities (reflected dichotomously in order to derive a parsimonious score, including coronary artery disease, heart failure, hypertension, chronic obstructive pulmonary disease, asthma, obstructive sleep apnea, chronic kidney disease, end-stage renal disease, diabetes, cirrhosis, cancer, and organ transplantation), and body mass index (BMI) (kg/m^2^). We extracted the patient’s most recent vital signs, including temperature (degrees Celsius), respiratory rate (breaths per minute), pulse rate (beats per minute), oxygen saturation (%), and systolic blood pressure (mmHg; but not diastolic blood pressure which would be colinear). We extracted common laboratory measures that had shown promise in prior case series, including creatinine (mg/dL), glucose (mg/dL), total bilirubin (mg/dL), and acute phase reactants including white blood cell count (K/μL), albumin (g/dL), lactate dehydrogenase (LDH) (U/L), high sensitivity troponin (ng/L), C-reactive protein (CRP) (mg/L), procalcitonin (ng/mL), and D-dimer (ng/mL). We did not include medication treatments given their unknown consequences.^20^

### Sample Size

Our dataset is comprised of a training cohort to derive a risk score and a validation cohort to validate the score. To ensure that the sample size in the training (derivation) cohort was sufficient for the estimates and for *p* values to be valid, we applied the rule that the number of events (positive for the composite outcome of hypoxia, ICU, or death) and non-events (negative for the composite) per covariate in the model should be at least 10.^21–23^ Given we categorized continuous variables into quartiles (for reasons discussed below), each of these predictors would act as three “covariates.” We aimed for a parsimonious model with no more than 5 predictors (and thus no more than 15 “covariates”). Thus, our derivation cohort would require at least 150 events and 150 non-events. With 1014 patients in the training cohort (759 events and 255 non-events), we had sufficient patients to validly estimate the beta coefficients in the logistic regression model.

### Statistical Analysis

We report descriptive statistics on all variables, reporting means and 95% confidence intervals or frequencies and percentages. The study sample was randomly divided into a 75% cohort for training and a 25% cohort for validation.^24, 25^

To facilitate creation of a “paper and pencil” risk score, we categorized continuous variables. In other circumstances, clinically relevant thresholds would be used to categorize variables. However, the uncertainty about the pathophysiology underlying rapid progression to acute respiratory distress syndrome and unusual clinical characteristics in general left us uncertain about appropriate thresholds; we therefore chose to categorize by each variable’s quartiles. We first examined bivariate comparisons of the categorized variables using a chi-squared test. We retained all variables with *p* < 0.1 for the manual forward selection process. We removed any variable with more than 10% missingness. A priori and to mimic the order in which data are typically available during an encounter, we performed manual stepwise forward logistic regression, beginning with age and sex, then vital signs, then comorbidities, and finally laboratory values. As noted above, to achieve a parsimonious model usable at the bedside, we decided a priori to include no more than 5 variables. We only added a variable if it made a more than negligible change to the model’s fit, determined by the *c*-statistic (estimate of the area under the receiver operator characteristic curve). We adjusted for clustering by hospital but found no difference in the model’s performance.

We derived the risk score’s points by comparing the *β* coefficient of the variable to the overall sum of coefficients in the model, multiplying by 100, and rounding to the nearest integer.^26^ The risk score calculated for each patient represented the summed point totals from all variables present, where a higher score indicates more suitability for discharge.

We calculated a risk score for each patient, and the optimal cut point in the risk score was chosen as the cut point that maximized the sum of the sensitivity and specificity for suitability for discharge.^27^ The *c*-statistic was used to assess the discriminative ability of the logistic regression model and the risk score in both the training and validation cohorts. We validated the risk score by calculating the *c*-statistic for the score’s ability to discriminate who is and is not suitable for discharge in the validation cohort (remaining 25% of the original sample). The Hosmer-Lemeshow goodness-of-fit statistic was used to assess calibration for the logistic regression model in both the training and validation cohorts. Anticipating the risk score’s real-world use, we also examined its discriminative capacity for inpatients versus emergency department patients by validating the model for subsets of the population. As an added test, we checked the interaction between a patient’s score and their site of care. Upon recommendation from our emergency medicine colleagues, we also tested a competing model without any laboratory values, presented in the Supplement.

All tests for significance used a 2-sided *p* value of 0.05 unless otherwise noted. We performed all analyses in SAS, version 9.4 (SAS Institute).

## RESULTS

### Patient Characteristics

**Figure 1:**
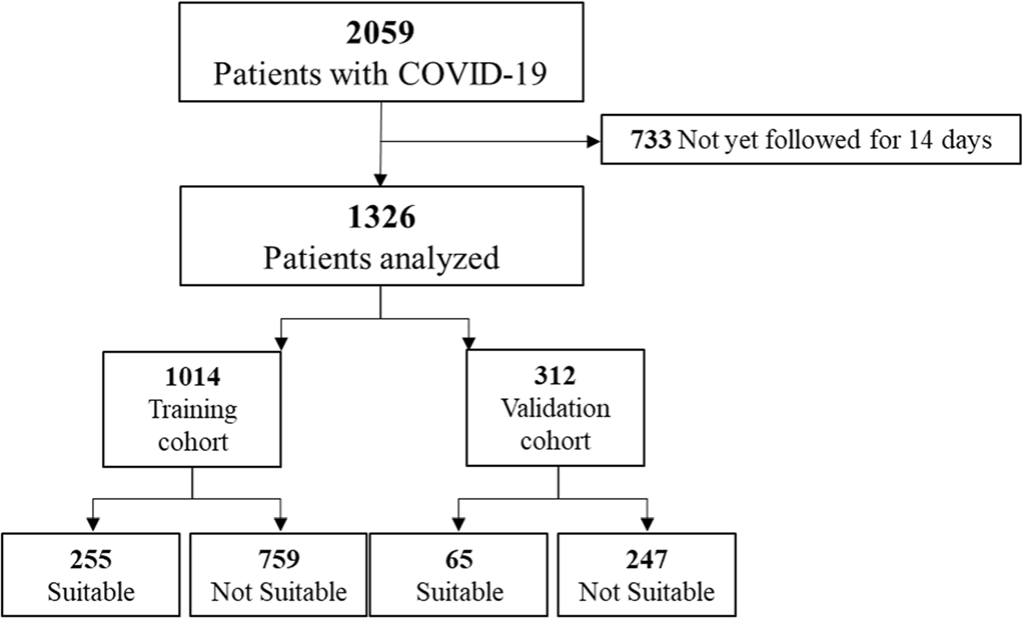
**Sample selection schematic. COVID-19, coronavirus disease 2019.**

A total of 2059 patients tested positive for SARS-CoV-2, 1326 met criteria for inclusion (733 did not have the necessary 14-day follow-up), and 1014 were included in the training cohort (Fig. 1). Patients in the training cohort had a mean age of 58 years (95% CI, 57 to 59) and were 56% male, 51% White, 41% employed, 43% privately insured, and 68% never-smokers (Table 1 and eTable 2). Most (65%) had at least one chronic condition, with hypertension (46%), diabetes (31%), chronic kidney disease (15%), and asthma (14%) the most common (eTable 3).

**Table 1:** Training Cohort Patient Characteristics and Bivariate Differences (the Table Shows the 75% (Training) Cohort; See eTable 2 for the 25% (Validation) Cohort)

Characteristic^**a**^	Training cohort (*n* = 1014)	Suitable for discharge		*p* value^**b**^
		Yes (*n* = 255)	No (*n* = 759)	
	*n* (%)			
Sociodemographics				
Age, years				
18–45	270 (27)	128 (50)	142 (19)	< 0.0001
46–59	241 (24)	68 (27)	173 (23)	
60–73	255 (25)	36 (14)	219 (29)	
> 73	248 (24)	23 (9)	225 (30)	
Sex				
Male	567 (56)	115 (45)	452 (60)	< 0.0001
Female	447 (44)	140 (55)	307 (40)	
Race/ethnicity				
White	513 (51)	132 (52)	381 (50)	0.5025
Black	113 (11)	33 (13)	80 (11)	
Latino	221 (22)	52 (20)	169 (22)	
Asian	37 (4)	12 (5)	25 (3)	
Other	37 (4)	8 (3)	29 (4)	
Unavailable	93 (9)		75 (10)	
Language				
English	659 (65)	187 (73)	472 (62)	0.005
Spanish	236 (23)	43 (17)	193 (25)	
Unknown	46 (5)	13 (5)	33 (4)	
Other	73 (7)	12 (5)	61 (8)	
Employment				
Employed	416 (41)	152 (60)	264 (35)	< 0.0001
Unemployed	212 (21)	42 (16)	170 (22)	
Retired	246 (24)	24 (9)	222 (29)	
Unknown	140 (14)	37 (15)	103 (14)	
Primary insurance				
Private	436 (43)	158 (62)	278 (37)	< 0.0001
Medicare	370 (36)	43 (17)	327 (43)	
Medicaid	164 (16)	34 (13)	130 (17)	
Uninsured	44 (4)	20 (8)	24 (3)	
Tobacco use				
Never-smoker	688 (68)	204 (80)	484 (64)	< 0.0001
Ever-smoker	326 (32)	51 (20)	275 (36)	
Comorbidities				
None	360 (36)	128 (50)	232 (31)	< 0.0001
Any^**c**^	654 (65)	127 (50)	527 (69)	
Body mass index, kg/m^2^				
< 25.6	238 (25)	65 (34)	173 (23)	0.0147
25.6–29.3	236 (25)	47 (24)	189 (25)	
29.4–33.7	237 (25)	38 (20)	199 (26)	
> 33.7	236 (25)	43 (22)	193 (26)	
Vital signs				
Temperature, °C				
< 36.4	294 (29)	63 (26)	231 (31)	0.0858
36.4–36.8	254 (25)	77 (31)	177 (23)	
36.9–37.2	247 (25)	58 (24)	189 (25)	
> 37.2	206 (21)	47 (19)	159 (21)	
Heart rate, beats per minute				
< 72	262 (26)	63 (26)	199 (27)	0.6625
72–81	234 (24)	63 (26)	171 (23)	
82–92	262 (26)	68 (28)	194 (26)	
> 92	237 (24)	53 (21)	184 (25)	
Respiratory rate, breaths per minute				
< 18	424 (43)	158 (66)	266 (36)	< 0.0001
18–20	234 (24)	57 (24)	177 (24)	
21–24	100 (10)	10 (4)	90 (12)	
> 24	226 (23)	13 (5)	213 (29)	
Systolic blood pressure, mmHg				
< 109	256 (26)	43 (17)	213 (28)	0.0069
109–120	253 (25)	70 (28)	183 (24)	
121–134	246 (25)	71 (29)	175 (23)	
> 134	247 (25)	62 (25)	185 (24)	
Oxygen saturation, %				
< 94	302 (30)	16 (7)	286 (38)	< 0.0001
94–96	275 (28)	50 (21)	225 (30)	
97–98	278 (28)	98 (40)	180 (24)	
> 98	142 (14)	79 (33)	63 (8)	
Laboratory measures				
Glucose, mg/dL				
< 98	243 (25)	85 (40)	158 (21)	< 0.0001
98–114	248 (26)	60 (28)	188 (25)	
115–150	239 (25)	45 (21)	194 (26)	
> 150	240 (25)	22 (10)	218 (29)	
Creatinine, mg/dL				
< 0.69	260 (27)	57 (27)	203 (27)	< 0.0001
0.69–0.87	230 (24)	68 (32)	162 (21)	
0.88–1.20	244 (25)	62 (29)	182 (24)	
> 1.20	237 (24)	25 (12)	212 (28)	
White blood cell count, K/μL				
< 5.2	245 (25)	66 (31)	179 (24)	< 0.0001
5.2–6.9	244 (25)	69 (32)	175 (23)	
7.0–9.5	243 (25)	51 (24)	192 (25)	
> 9.5	243 (25)	30 (14)	213 (28)	
Albumin, g/dL				
< 2.8	245 (26)	7 (4)	238 (31)	< 0.0001
2.8–3.3	262 (27)	13 (7)	249 (33)	
3.4–3.7	211 (22)	37 (19)	174 (23)	
> 3.7	238 (25)	143 (72)	95 (13)	
Bilirubin, mg/dL				
< 0.3	304 (33)	77 (39)	227 (31)	0.0296
0.3–0.4	200 (22)	40 (21)	160 (22)	
0.5–0.6	218 (23)	48 (25)	170 (23)	
> 0.6	207 (22)	30 (15)	177 (24)	

^a^Any measure missing more than 10% of data was a priori not included in the regression model. See eTable 4 for detail on laboratory values not included

^b^Chi-square test between suitable and not suitable

^c^See eTable 3 for detail on comorbidities

Of the patients in the training cohort, 255 (25%) were suitable for discharge, 783 (77%) were admitted, 728 (72%) required oxygen, 388 (38%) required ICU care, and 55 (5%) expired.

About a quarter of patients’ most recent vital signs were potentially concerning (Table 1). Specifically, 237 (24%) had a heart rate greater than 92, 226 (23%) had a respiratory rate greater than 24, 256 (26%) had a systolic blood pressure less than 109, and 302 (30%) had oxygen saturation less than 94%. A large percentage of patients had aberrations in acute phase reactants; for example, 245 (26%) had albumin less than 2.8 g/dL and 203 (25%) had LDH greater than 387 U/L (eTable 4).

### Risk Score Development

**Figure 2:**
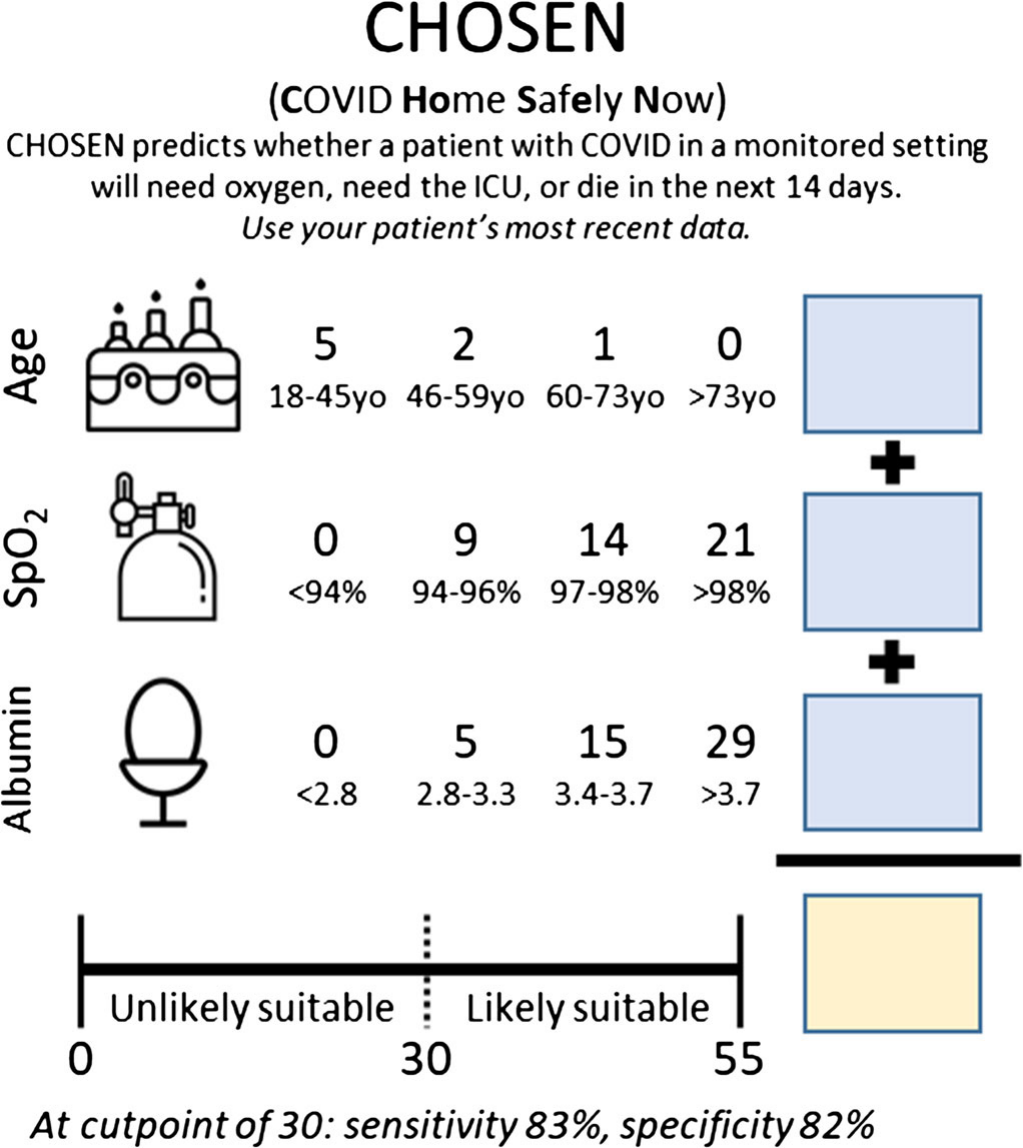
**Risk score tool for the point of care.**

Of the characteristics in Table 1, all except race/ethnicity (*p* = 0.50) and heart rate (*p* = 0.66) were significant in bivariate analysis. We added variables in a prespecified manual forward stepwise manner (eTable 5). The final model included age, oxygen saturation, and albumin, resulting in a score between 0 and 55 (Table 2; Fig. 2). Albumin was the most predictive factor (aOR of albumin > 3.7 g/dL compared to < 2.8 g/dL, 42 [95% CI, 18 to 96]). Age was the least (aOR of age 18–45 compared to age > 73, 1.8 [95% CI, 0.9 to 3.4]). The risk score had good discriminative capacity (*c*-statistic, 0.89 [95% CI, 0.87 to 0.91]) and no evidence of lack of fit (*p* = 0.835). At a cut point of 30, it had a sensitivity of 83.2%, and a specificity of 82.2% (Table 3). A false positive rate of 17.8 demonstrated a conservative score. We also considered other models with respiratory rate and without laboratory values, discussed below and in the online supplement.

**Table 2:** Multivariable Model and Associated Risk Score^a^

Characteristic	Adjusted odds ratio (95% CI)	Score
Age, years		
18–45	1.8 (0.9, 3.4)	5
46–59	1.3 (0.7, 2.6)	2
60–73	1.1 (0.5, 2.1)	1
> 73	1 (Reference)	0
Oxygen saturation, %		
< 94	1 (Reference)	0
94–96	3.2 (1.6, 6.3)	9
97–98	6.4 (3.3, 12.3)	14
>98	14.9 (7.1, 31.4)	21
Albumin, g/dL		
< 2.8	1 (Reference)	0
2.8–3.3	1.9 (0.7, 4.9)	5
3.4–3.7	6.8 (2.9, 16.1)	15
> 3.7	41.7 (18.1, 96.4)	29

*c*-statistic: 0.8939 (95% CI, 0.8687 to 0.9192)

^**a**^See eTable 5 for Description of the Forward Step Method That Resulted in the Above Model; See eTable 6 for a Model Not Requiring Laboratory Values and eTable 7 for a Model with Respiratory Rate

**Table 3:** Risk Score Performance

Score	Sensitivity	Specificity	False positive	False negative
0	100	9.2	90.8	0
5	98.5	15.1	84.9	1.5
10	98	32.4	67.6	2
15	97.4	43.4	56.6	2.6
20	94.9	61.6	38.4	5.1
25	88.8	74.2	25.8	11.2
30	83.2	82.2	17.8	16.8
35	70.9	90.1	9.9	29.1
40	63.3	93.6	6.4	36.7
45	50	96.4	3.6	50
50	23	99.2	0.8	77
55	10.7	99.7	0.3	89.3

A higher score indicates greater suitability for discharge. Score of 30 optimized sensitivity and specificity. Clinicians can choose a cut point that reflects their clinical milieu. For example, if beds and resources are freely available, one might choose a higher cut point. “False positive” indicates the risk score would trigger as not suitable for discharge when in fact the patient was suitable. “False negative” indicates the risk score would trigger as suitable for discharge when in fact the patient was not suitable

### Risk Score Validation

In the validation cohort, there was no significant difference in the model’s discriminative capacity (*c*-statistic, 0.87 [95% CI, 0.81 to 0.93]) and no evidence of lack of fit (*p* = 0.435) (eTable 6; eFigure 1).

To ensure the risk score performed well for both inpatients and other patients, we also stratified the validation by inpatients and emergency department patients. There were no significant differences in model performance with respect to the *c*-statistic, although the model performed somewhat better in the emergency department (eTable 6). The relationship between the composite outcome and the score was similar for inpatients and emergency department patients; in particular, the interaction of score with inpatient status in a logistic regression model to predict the composite outcome was not significant for the training (*p* for the interaction effect, 0.772) or validation cohort (*p* for the interaction effect, 0.2772).

## DISCUSSION

We developed and validated a 3-item risk score to be used at the point of care to assist clinicians in decisions regarding discharge from a monitored setting for patients with COVID-19 (Fig. 2). The simple score, CHOSEN (**C**OVID **Ho**me **S**af**e**ly **N**ow), has robust discrimination, sensitivity, and specificity across all patients with COVID-19 in a monitored setting.

With over 600,000 infections in the USA, many hospital systems are severely strained. In the majority of cases, the patient appears clinically stable, an instance where CHOSEN could be useful. Currently, physicians are faced with a lack of validated risk scores to aid bedside management. Our risk score builds on important work from the Brescia-COVID Respiratory Severity Scale, which is appropriate for patients in respiratory distress who must be re-evaluated every few hours or even every few minutes.^28^ Shi et al. have described a “host susceptibility score” that assigns one point each for age ≥ 50, male sex, and presence of hypertension, and that predicts death or a severe phenotype.^29^ The unclear definition of the endpoint, absence of information about missing data, and lack of description of model-building methodology make it difficult to ascertain why their results were different from ours. Also, this and several other studies lack a standardized follow-up period, which could lead to biased estimates and incorrect conclusions.^30^

Our work also builds upon several case series that have examined patient characteristics associated with various negative outcomes. These case series collected clinical data and laboratory values to identify risk factors linked to the progression of patients with COVID-19.^3–5, 19^ Older age has been found to be a risk factor, but there is no clear threshold below which age is protective nor comparison of age to other clinical criteria. In contrast, we found age was much less predictive than other factors. Several authors have linked comorbidities with poor outcomes.^3, 7^ However, comorbidities were not universal among seriously ill patients^3^ and in our multivariable model, the presence of a comorbidity was not significant. Our work concurs with others regarding acute phase reactants. We found albumin, a negative acute phase reactant, as highly predictive, in concert with a smaller case series in a group of patients with progressive disease.^7, 30, 31^

Available data suggest that COVID-19 represents a diversion from the typical viral pneumonia clinical course such that it often initially presents without clear signs of decompensation, only to later be catastrophic. In support of this, we saw relatively narrow quartiles in the included variables, which speaks to the subtle, but important, changes in clinical presentation that occur with COVID-19. These highly nuanced changes may not be immediately discernable to a clinician in the moment, making a risk score like CHOSEN clinically useful.

We encountered challenges when trying to include respiratory rate, an often subjectively measured variable with significant measurement error.^32^ Given this known issue, it is not surprising that we were unable to divide patients evenly into quartiles; all modelers should approach this variable with caution (Table 1). We therefore present the more parsimonious and similarly performing model without respiratory rate (Fig. 2) but include a competing model with respiratory rate in the online supplement for clinicians to use at their own discretion (eTables 7–9; eFigure 2). We also include a competing model without any laboratory values based on feedback from emergency medicine colleagues, although this model did not perform as well (eTable 10).

Defining the outcome’s follow-up period was challenging due to variable findings in the literature regarding disease course. Our follow-up period of at least 14 days may be insufficient. We considered a longer follow-up period but balanced the advantages of that with the goal of delivering a useful tool to clinicians as soon as possible. However, by defining day zero as a time point later than symptom onset (our day zero was date of positive test or date of presentation) and including events upstream of death (any oxygen requirement and ICU-level care), we felt 14 days was a suitable time period to capture the majority of events. The model may not perform well for patients who deteriorate after 14 days, but in times of extreme resource scarcity, it may be necessary for those patients to convalesce at home until they require hospital-level care. We also recognize that some institutions are discharging patients with COVID-19 on oxygen, and our model does not account for this as a disposition option. The 14-day follow-up period explains why 733 patients with COVID-19 were not included in the analyzed sample, a relatively large number because testing was ramping up during this period.

Our study has limitations. First, our data source was the EHR, which has known data quality issues including inaccurate coding of comorbidities. To mitigate this, we pulled not only from a patient’s problem list but also from disease registries. Also, in bedside management, a patient’s problem list is often what is quickly available to a clinician. We were not able to pull every a priori risk factor (e.g., ferritin). However, prior work has noted significant correlation among acute phase reactants, such that one is likely sufficient (e.g., albumin).^7^ We similarly were unable to extract unstructured data, such as date of symptom onset. Second, a patient may have required care at a facility outside of Mass General Brigham after first presenting for testing, thereby limiting our ability to capture the composite endpoint and misclassifying the patient as “suitable for discharge.” However, in our prior studies using the same EHR with phone calls and a regional health information network to confirm utilization, we found that acute care at an outside system following discharge was exceedingly low.^33^ Third, although possibly uncommon, a family could have expressed the desire for discharge to home with an advanced directive specifying home-first palliative care. In this case, the patient would have passed away and been counted as having reached the composite endpoint, though this is the optimal outcome in these cases. Finally, our sample is from a single region in New England and was early in the pandemic where limited testing and extreme pressures on bed capacity existed. This may therefore not be generalizable to other populations or later periods of the pandemic, although similar hotspots have occurred throughout the country and globe. Similarly, we note that triage decision-making has evolved, and local practice variation exists based on system pressures and discharge resources (e.g., supplemental oxygen or remote monitoring at home).^34^ CHOSEN may offer a guidepost for which clinicians should account for their local system pressures and discharge resources when making discharge decisions. We plan for a prospective evaluation of this risk score to ensure validity and external validation in other populations and time periods to ensure generalizability, as derivation models may result in overly optimistic results compared with external validation cohorts.

## CONCLUSIONS

A simple point of care 3-item risk score that includes age, oxygen saturation, and albumin predicts with good discriminative capacity, sensitivity, and specificity whether a patient with COVID-19 is suitable for discharge.

## Supplementary Information

ESM 1 (DOCX 116 kb)
