# Supplementary material for: Derivation of a Clinical Risk Score to Predict 14-Day Occurrence of Hypoxia, ICU Admission, and Death Among Patients with Coronavirus Disease 2019
Source: J Gen Intern Med. 2020 Dec 3;36(3):730–7. doi: 10.1007/s11606-020-06353-5 (PMC7713904; doi:10.1007/s11606-020-06353-5)

Derivation of a clinical risk score to predict 14-day occurrence of hypoxia, ICU admission, and death among patients with Coronavirus Disease 2019

**Supplemental Material**

***Corresponding Author:**

David Michael Levine

Harvard Medical School

Brigham and Women’s Hospital

Division of General Internal Medicine and Primary Care

1620 Tremont Street, 3rd floor

Boston, MA 02120

Email: dmlevine@bwh.harvard.edu

Phone, office: 617.732.8925

Phone, cell: 847.400.4211

Fax: 617.732.7072

**eTable 1**. Evolution of testing criteria at Mass General Brigham institutions

| Date | Criteria |
| --- | --- |
| March 1 – March 15, 2020 | To be tested, you must have at least one of the following, potentially attributable to a respiratory viral illness:  1. Fever  2. Sore Throat  3. Cough  4. Shortness of Breath  5. Muscle aches  And at least one of the following:  1. Inpatients  2. Emergency department patient requiring admission  3. Partners healthcare worker who currently works at a clinical site and who worked while sick  4. Patient cared for by symptomatic Partners healthcare worker with confirmed COVID19 |
| March 16, 2020 | ***Addition of*** ED high risk* patients not requiring admission |
|  | **High Risk is defined as:**  1. Age ≥70  2. Severe chronic lung disease (e.g. asthma, bronchiectasis, cystic fibrosis, COPD, etc.)  3. Severe heart disease  4. CD4 count <200  5. On immunocompromising medications (e.g. prednisone >20mg/d, chemotherapy, mycophenolate, cyclosporine, azathioprine, tacrolimus, TNF inhibitors, monoclonal antibodies, etc.) |
| March 17, 2020 | ***Addition of* Asymptomatic:**  COVID-19 positive healthcare worker, now asymptomatic, requiring return to work clearance |
|  | ***Addition of* Symptomatic:**  1. Household contacts of a Partners Healthcare employee who is required to work at a clinical site  2. Retest patients with indeterminate/inconclusive COVID-19 result  3. COVID-19 positive healthcare workers who require clearance prior to returning to work, per OHS policy  4. Homeless individuals regardless of whether or not they are part of a cluster or had contact with a confirmed case. |
|  | **High risk definition expanded to:**  1. Dialysis  2. Long term care facility or group home setting.  3. Homeless |
| March 19, 2020 | ***Addition of* Asymptomatic**  Organ Donor |
|  | ***Addition of* Symptomatic**  High risk outpatient seen in Respiratory Illness Clinic |
| March 20,2020 | ***Addition of* Asymptomatic:**  Patients presenting in extremis and no history able to be obtained |
|  | ***Addition of* Symptomatic:**  Patients receiving dialysis moved from high risk definition to symptomatic category |
| March 21, 2020 | ***Addition of* Asymptomatic:**  Retest patients with indeterminate/inconclusive COVID-19 result moved from symptomatic to asymptomatic |
| March 22, 2020 | **Asymptomatic**  1. Patients undergoing urgent airway surgery in the next 48 hours that cannot be deferred  2. Patients requiring urgent transplants within the next 48 hours  (solid or liquid) |
|  | **High risk definition refined to:**  1. Severe chronic lung disease (e.g. asthma, bronchiectasis, cystic fibrosis, COPD, bronchopulmonary dysplasia, Cerebral Palsy with recurrent pneumonia, trach dependency, etc.)  2. Severe heart disease (including congenital heart disease) |
| March 24, 2020 | ***List of symptoms expanded to include:***  1. Runny nose or nasal congestion  2. Anosmia |
| March 26, 2020 | **Symptomatic:**  ED patients, regardless of need for admission |
| March 27,2020 | **Asymptomatic:**  COVID-19 inpatients who meet Partners criteria for resolution of infection status |
| March 31, 2020 | **Asymptomatic:**    1. Patients requiring urgent transplants or immune suppression within the next 48-hours (solid organ transplant, BMT, CAR-T, and leukemia induction/consolidation or other high-intensity chemotherapy)  2. Patients ready for discharge where post-acute care facility requires negative test |
| April 1, 2020 | **Symptomatic:**  Women in late pregnancy (>=36 weeks) |
| April 2, 2020 | **Symptomatic:**  Expanded to all high-risk outpatients |
| April 3, 2020 | **Asymptomatic**  Neonates born to confirmed COVID-19+ mothers |
| April 4, 2020 | **Asymptomatic**  Patients being admitted from a skilled nursing facility or other long-term care facility |
| April 8, 2020 | **Asymptomatic**  1. Patients being admitted to a Partners facility from a skilled nursing facility, psychiatric, substance abuse, or rehabilitation hospital, hospice, or correctional facility  2. COVID-19 positive outpatients who require testing for resolution of infection status per Partners Policy due to urgent need for in-person care within the next 30 days |
|  | **High risk definition refined to:**  Residents of a long-term care facility or group home setting (testing to be done at a Partners testing site) |
| April 10, 2020 | **Asymptomatic:**  Patients being admitted to a Partners inpatient psychiatric facility at time of admission. |
| April 11, 2020 | **Symptomatic:**  High risk outpatient seen in Massachusetts General Hospital-Chelsea Respiratory Illness Clinic |
|  | **High risk definition expanded to:**  Residents of Chelsea, MA |

**eTable 2**. Validation cohort patient characteristics

|  |  | Suitable for discharge | |
| --- | --- | --- | --- |
| Characteristic**^b^** | Validation cohort (n=312) | Yes (n=65) | No (n=247) |
|  | n (%) | | |
| **Sociodemographics** | | | |
| Age, years |  |  |  |
| 18-47 | 76 (24) | 28 (43) | 48(19) |
| 48-63 | 57 (18) | 11 (17) | 46 (19) |
| 64-78 | 80 (26) | 16 (26) | 64 (26) |
| >78 | 99 (32) | 10 (15) | 89 (36) |
| Sex |  |  |  |
| Male | 165 (53) | 30 (46) | 135 (55) |
| Female | 147 (47) | 35 (54) | 112 (45) |
| Race/ethnicity |  |  |  |
| White | 173 (55) | 44 (68) | 129 (52) |
| Black | 31 (10) | 6 (9) | 25 (10) |
| Latino | 64 (21) | 8 (12) | 56 (23) |
| Asian | 10 (3) | 4 (6) | 6 (2) |
| Other | 6 (2) | 1 (2) | 5 (2) |
| Unavailable | 28 (9) | 2 (3) | 26 (11) |
| Language |  |  |  |
| English | 205 (66) | 51 (78) | 154 (62) |
| Spanish | 79 (25) | 9 (14) | 70 (28) |
| Unknown | 11 (4) | 2 (3) | 9 (4) |
| Other | 17 (5) | 3 (5) | 14 (6) |
| Employment |  |  |  |
| Employed | 106 (34) | 35 (54) | 71 (29) |
| Unemployed | 55 (18) | 8 (12) | 47 (19) |
| Retired | 96 (31) | 12 (19) | 84 (34) |
| Unknown | 55 (18) | 10 (15) | 45 (18) |
| Primary Insurance |  |  |  |
| Private | 128 (41) | 39 (60) | 89 (36) |
| Medicare | 131 (42) | 18 (28) | 113 (46) |
| Medicaid | 44 (14) | 7 (11) | 37 (15) |
| Uninsured | 9 (3) | 1 (2) | 8 (3) |
| Tobacco Use |  |  |  |
| Never-smoker | 209 (67) | 47 (72) | 162 (66) |
| Ever-smoker | 103 (33) | 18 (28) | 85 (34) |
| Comorbidities |  |  |  |
| None | 107 (34) | 31 (48) | 76 (31) |
| Any | 205 (66) | 34 (52) | 171 (69) |
| Body Mass Index, kg/m^2^ |  |  |  |
| <25.8 | 65 (22) | 13 (28) | 52 (21) |
| 25.8 – 29.1 | 87 (30) | 8 (17) | 79 (32) |
| 29.2 – 34.2 | 60 (20) | 10 (21) | 50 (20) |
| >34.2 | 82 (28) | 16 (34) | 66 (27) |
| **Vital Signs** | | | |
| Temperature, ºC |  |  |  |
| <36.3 | 97 (32) | 18 (30) | 79 (32) |
| 36.3 – 36.8 | 69 (22) | 20 (33) | 49 (20) |
| 36.9 – 37.2 | 70 (23) | 13 (21) | 57 (23) |
| >37.2 | 71 (23) | 10 (16) | 61 (25) |
| Heart rate, beats per minute |  |  |  |
| <71 | 86 (29) | 15 (24) | 71 (30) |
| 71-81 | 63 (21) | 12 (19) | 51 (21) |
| 82-94 | 73 (24) | 17 (27) | 56 (23) |
| >94 | 80 (27) | 18 (29) | 62 (26) |
| Respiratory rate, breaths per minute |  |  |  |
| <18 | 121 (40) | 40 (69) | 81 (34) |
| 18-20 | 80 (27) | 11 (19) | 69 (29) |
| 21-24 | 27 (9) | 3 (5) | 24 (10) |
| >24 | 72 (24) | 4 (7) | 68 (28) |
| Systolic blood pressure, mmHg |  |  |  |
| <111 | 62 (20) | 10 (16) | 52 (21) |
| 111-123 | 74 (24) | 13 (21) | 61 (25) |
| 124-138 | 80 (26) | 14 (23) | 66 (27) |
| >138 | 90 (29) | 24 (39) | 66 (27) |
| Oxygen saturation, % |  |  |  |
| <94 | 103 (34) | 5 (8) | 98 (40) |
| 94-86 | 84 (28) | 16 (27) | 68 (28) |
| 97-98 | 76 (25) | 26 (43) | 50 (20) |
| >98 | 43 (14) | 13 (22) | 30 (12) |
| **Laboratory Measures** | | | |
| Glucose, mg/dL |  |  |  |
| <98.5 | 75 (25) | 18 (34) | 57 (23) |
| 98.5-115 | 69 (23) | 17 (32) | 52 (21) |
| 116-153.5 | 70 (23) | 15 (28) | 55 (22) |
| >153.5 | 86 (29) | 3 (6) | 83 (34) |
| Creatinine, mg/dL |  |  |  |
| <0.68 | 80 (27) | 13 (24) | 67 (27) |
| 0.68-0.86 | 77 (26) | 15 (28) | 62 (25) |
| 0.87-1.20 | 69 (23) | 19 (35) | 50 (20) |
| >1.20 | 75 (25) | 7 (13) | 68 (28) |
| White blood cell count, K/uL |  |  |  |
| <5.4 | 69 (23) | 15 (27) | 54 (22) |
| 5.4-7.0 | 79 (26) | 12 (22) | 67 (27) |
| 7.1-9.2 | 83 (28) | 16 (29) | 67 (27) |
| >9.2 | 70 (23) | 12 (22) | 58 (24) |
| Albumin, g/dL |  |  |  |
| <2.8 | 91 (30) | 2 (4) | 89 (36) |
| 2.8-3.3 | 71 (24) | 6 (11) | 65 (26) |
| 3.4-3.6 | 75 (25) | 9 (17) | 66 (27) |
| >3.6 | 62 (21) | 36 (68) | 26 (11) |
| Bilirubin, mg/dL |  |  |  |
| <0.3 | 85 (29) | 13 (27) | 72 (30) |
| 0.3-0.5 | 56 (19) | 6 (12) | 50 (21) |
| 0.5-0.6 | 78 (27) | 13 (27) | 65 (27) |
| >0.6 | 72 (25) | 17 (35) | 55 (23) |

**^a^**: See **Table 1** for the 75% (training) sample.

**^b^**: Any measure missing more than 10% of data was *a priori* not included.

**eTable 3**. Training cohort detailed comorbidities

|  |  | Suitable for discharge | |
| --- | --- | --- | --- |
| Characteristic | Training cohort (n=1014) | Yes (n=255) | No (n=759) |
|  | n (%) | | |
| Coronary artery disease |  |  |  |
| No | 924 (91) | 242 (95) | 682 (90) |
| Yes | 90 (9) | 13 (5) | 77 (10) |
| Heart failure |  |  |  |
| No | 909 (90) | 245 (96) | 664 (87) |
| Yes | 105 (10) | 10 (4) | 95 (13) |
| Hypertension |  |  |  |
| No | 545 (54) | 178 (70) | 367 (48) |
| Yes | 469 (46) | 77 (30) | 392 (52) |
| Chronic obstructive pulmonary disease |  |  |  |
| No | 941 (93) | 247 (97) | 694 (91) |
| Yes | 73 (7) | 8 (3) | 65 (9) |
| Asthma |  |  |  |
| No | 877 (86) | 213 (84) | 664 (87) |
| Yes | 137 (14) | 42 (16) | 95 (13) |
| Obstructive sleep apnea |  |  |  |
| No | 933 (92) | 238 (93) | 695 (92) |
| Yes | 81 (8) | 17 (7) | 64 (8) |
| Chronic kidney disease |  |  |  |
| No | 866 (85) | 238 (93) | 628 (83) |
| Yes | 148 (15) | 17 (7) | 131 (17) |
| End-stage renal disease |  |  |  |
| No | 992 (98) | 251 (98) | 741 (98) |
| Yes | 22 (2) | 4 (2) | 18 (2) |
| Diabetes |  |  |  |
| No | 703 (69) | 207 (81) | 496 (65) |
| Yes | 311 (31) | 48 (19) | 263 (35) |
| Cirrhosis |  |  |  |
| No | 1002 (99) | 253 (99) | 749 (99) |
| Yes | 12 (1) | 2 (1) | 10 (1) |
| Cancer |  |  |  |
| No | 885 (87) | 228 (89) | 657 (87) |
| Yes | 129 (13) | 27 (11) | 102 (13) |
| Transplant |  |  |  |
| No | 995 (98) | 251 (98) | 657 (87) |
| Yes | 19 (2) | 4 (2) | 102 (13) |

**Note**: comorbidities were captured through a patient’s electronic health record’s problem list and population health disease registries.

**eTable 4**. Clinical characteristics removed *a priori* due to missingness of more than 15%**^a^**

|  |  | | Suitable for discharge | | | |
| --- | --- | --- | --- | --- | --- | --- |
| Characteristic | Training cohort (n=1014) | | Yes (n=255) | | No (n=759) | |
|  | n | mean (95% CI) | n | mean (95% CI) | n | mean (95% CI) |
| C-reactive protein, mg/L | 138 | 79.0 (65.8,92.1) | 12 | 49.4 (-0.9,99.8) | 126 | 81.8 (68.1,95.5) |
| D-dimer, ng/mL | 631 | 1677 (1543,1812) | 56 | 855 (628,1082) | 575 | 1757 (1613,1902) |
| Lactate dehydrogenase, U/L | 818 | 334 (320,348) | 92 | 242 (225,258) | 726 | 346 (330,361) |
| Procalcitonin, ng/mL | 774 | 0.8 (0.5,1.1) | 71 | 0.1 (0.1,0.2) | 703 | 0.9 (0.6,1.2) |
| Troponin, ng/L | 421 | 56.1 (21.7,90.5) | 1 | 33.0 (n/a) | 420 | 56.2 (21.7,90.6) |

**^a^:** This table reflects the training sample.

**eTable 5**. Forward step logistic regression details

| Step | c-statistic | Action |
| --- | --- | --- |
| Start with age | 0.7170 | Include |
| Add sex | 0.7289 | Do not include |
| Add spo2 | 0.7995 | Include |
| Add systolic blood pressure | 0.8115 | Do not include |
| Add temperature | 0.8012 | Do not include |
| Add any comorbidity | 0.8002 | Do not include |
| Add smoking status | 0.8005 | Do not include |
| Add body mass index | 0.7831 | Do not include |
| Add glucose | 0.8022 | Do not include |
| Add creatinine | 0.7909 | Do not include |
| Add white blood cell count | 0.8017 | Do not include |
| Add albumin | 0.8939 | Include**^a^** |
| Add bilirubin | 0.8973 | Do not include |
| Add language | 0.8971 | Do not include |
| Add employment | 0.8950 | Do not include |
| Add insurance | 0.8941 | Do not include |

**^a^**: Hosmer-Lemeshow, p-value 0.8350

**eTable 6**. Training, validation, and inpatient vs emergency department analysis model outcomes

| Model | Cohort | Model | c-statistic (95% CI) |
| --- | --- | --- | --- |
| Age, oxygen saturation, albumin | Training | Regressed on model variables | 0.8939 (0.8687, 0.9192) |
|  | Validation | Regressed on model variables | 0.8685 (0.8095, 0.9275) |
|  | Validation | Regressed on score | 0.8701 (0.8121, 0.9280) |
|  | Validation | Regressed on model variables for inpatients | 0.7400 (0.5948, 0.8852) |
|  | Validation | Regressed on model variables for emergency department patients | 0.9214 (0.8172, 1.0000) |
|  | Validation | Regressed on score for inpatients | 0.7451 (0.6053, 0.8849) |
|  | Validation | Regressed on score for emergency department patients | 0.9214 (0.8172, 1.0000) |

**eTable 7**. Multivariable model and associated risk score with respiratory rate**^a^**

| Characteristic | Adjusted odds ratio (95% CI) | Score |
| --- | --- | --- |
| Age, years |  |  |
| 18-45 | 1.7 (0.9,3.4) | 4 |
| 46-59 | 1.3 (0.7,2.6) | 2 |
| 60-73 | 1.1 (0.6,2.3) | 1 |
| >73 | 1 [Reference] | 0 |
| Respiratory rate, breaths per minute |  |  |
| <18 | 4.1 (1.9,8.5) | 9 |
| 18-20 | 3.1 (1.4,6.8) | 8 |
| 21-24 | 1.6 (0.6,4.4) | 3 |
| >24 | 1 [Reference] | 0 |
| Oxygen saturation, % |  |  |
| <94 | 1 [Reference] | 0 |
| 94-96 | 3 (1.5,6) | 7 |
| 97-98 | 5.7 (2.9,11) | 12 |
| >98 | 12.4 (5.8,26.3) | 17 |
| Albumin, g/dL |  |  |
| <2.8 | 1 [Reference] | 0 |
| 2.8-3.3 | 1.6 (0.6,4.1) | 3 |
| 3.4-3.7 | 5.3 (2.2,12.7) | 11 |
| >3.7 | 30.1 (12.9,70.3) | 23 |

**^a^**: See **eTable 8** for description of the forward step method that resulted in the above model and **eTable 9** for the model’s performance. See **eTable 10** for a model and associated risk score not requiring laboratory values. See **Table 2** for a model without respiratory rate.

**eTable 8**. Forward step logistic regression details with respiratory rate

| Step | c-statistic | Action |
| --- | --- | --- |
| Start with age | 0.7170 | Include |
| Add sex | 0.7289 | Do not include |
| Add spo2 | 0.7995 | Include |
| Add systolic blood pressure | 0.8115 | Do not include |
| Add temperature | 0.8012 | Do not include |
| Add respiratory rate | 0.8322 | Include |
| Add any comorbidity | 0.8324 | Do not include |
| Add smoking status | 0.8334 | Do not include |
| Add body mass index | 0.8224 | Do not include |
| Add glucose | 0.8286 | Do not include |
| Add creatinine | 0.8227 | Do not include |
| Add white blood cell count | 0.8293 | Do not include |
| Add albumin | 0.9026 | Include**^a^** |
| Add bilirubin | 0.9046 | Do not include |
| Add language | 0.9040 | Do not include |
| Add employment | 0.9029 | Do not include |
| Add insurance | 0.9027 | Do not include |

**^a^**: Hosmer-Lemeshow, p-value 0.6792

Note the following operating characteristics:

| Model | Cohort | c-statistic (95% CI) |
| --- | --- | --- |
| Age, oxygen saturation, respiratory rate, albumin | Training, regressed on model variables | 0.9026 (0.8786, 0.9265) |
|  | Validation, regressed on model variables | 0.8890 (0.8379, 0.9401) |
|  | Validation, regressed on score | 0.8855 (0.8326, 0.9384) |

**eTable 9.** Risk score performance with respiratory rate

| Score | Sensitivity | Specificity | False Positive | False Negative |
| --- | --- | --- | --- | --- |
| 0 | 100 | 0 | 100 | 0 |
| 5 | 100 | 9.2 | 90.8 | 0 |
| 10 | 98.5 | 18.4 | 81.6 | 1.5 |
| 15 | 98 | 34.7 | 65.3 | 2 |
| 20 | 95.9 | 50.3 | 49.7 | 4.1 |
| 25 | 90.8 | 71.1 | 28.9 | 9.2 |
| 30 | 83.2 | 80.5 | 19.5 | 16.8 |
| 32 | 78.6 | 84.2 | 15.8 | 21.4 |
| 35 | 70.9 | 89.3 | 10.7 | 29.1 |
| 40 | 60.2 | 93.6 | 6.4 | 39.8 |
| 45 | 42.3 | 96.6 | 3.4 | 57.7 |
| 50 | 19.9 | 99.3 | 0.7 | 80.1 |
| 53 | 0 | 99.7 | 0.3 | 100 |

**Note:** a higher score indicates greater suitability for discharge.

**eTable 10.** Multivariable model and associated risk score without laboratory values**^a^**

| Characteristic | Adjusted odds ratio (95% CI) | Score |
| --- | --- | --- |
| Age, years |  |  |
| 18-45 | 5.0 (2.9,8.7) | 12 |
| 46-59 | 2.7 (1.6,4.8) | 8 |
| 60-73 | 1.5 (0.8,2.7) | 3 |
| >73 | 1 [Reference] | 0 |
| Respiratory rate, breaths per minute |  |  |
| <18 | 7.8 (4.2,14.6) | 16 |
| 18-20 | 5.1 (2.6,10.0) | 12 |
| 21-24 | 2.0 (0.8,4.9) | 5 |
| >24 | 1 [Reference] | 0 |
| Oxygen saturation, % |  |  |
| <94 | 1 [Reference] | 0 |
| 94-96 | 3.1 (1.7,5.8) | 9 |
| 97-98 | 7.4 (4.1,13.3) | 15 |
| >98 | 13.8 (7.3,26.3) | 20 |

**^a^**: See **Table 2** for model with laboratory values without respiratory rate. See **eTable 7** for a model with respiratory rate and laboratory values.

**Note**: c-statistic is 0.8322 (95% CI, 0.8021 to 0.8624). At a cut-point of 19 points (>19 likely suitable for discharge; range: 0-48), sensitivity is 72.2 and specificity is 73.2.

**eFigure 1.** Receiver operator characteristic curve


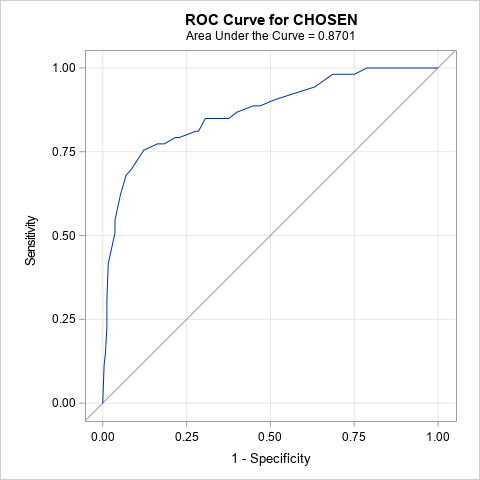


**eFigure 2.** Risk score tool for the point of care with respiratory rate


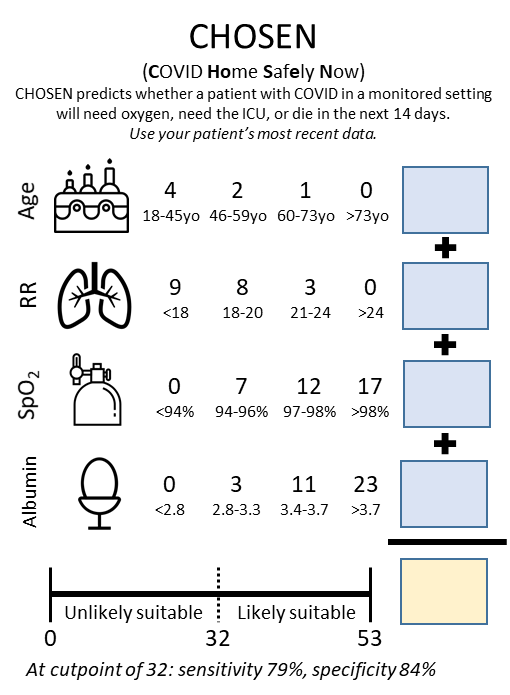

Supplement: Supplementary file 1 — (DOCX 116 kb) [file 11606_2020_6353_MOESM1_ESM.docx]
